# From fluoroscopy to artificial intelligence: three decades of percutaneous pelvic ring fixation—a bibliometric and science-mapping analysis (1996–2026)

**DOI:** 10.1007/s00402-026-06562-3

**Published:** 2026-11-04

**Authors:** Giovanni Vicenti, Davide Ferorelli, Michele Loiodice, Gianmarco Sirago, Giuseppe Solarino, Alessandro Dell’Erba

**Affiliations:** 1 Orthopaedic and Trauma Unit, Department of Translational Biomedicine and Neuroscience (DiBraiN), AOU Consorziale Policlinico, University of Bari Aldo Moro, Piazza Giulio Cesare 11, Bari, Italy; 2 Section of Legal Medicine, AOU Consorziale Policlinico, University of Bari Aldo Moro, Piazza Giulio Cesare 11, Bari, Italy

**Keywords:** Iliosacral screw, Surgical navigation, Robotics, Bibliometrics

## Abstract

**Introduction:**

Percutaneous pelvic-ring fixation requires implant placement within narrow osseous corridors. Navigation, robotics and artificial intelligence (AI) have successively been proposed to improve accuracy and reduce radiation exposure. This study mapped three decades of research on these technologies, characterising publication trends and the field’s intellectual, conceptual and social structure.

**Materials and methods:**

The Web of Science Core Collection (primary dataset, with parsed cited references) and Scopus (robustness comparison) were searched on 8 June 2026. Searches combined pelvic-fixation and technology terms and targeted articles, reviews and conference papers; degenerative-spine, deformity, arthroplasty and isolated acetabular records were excluded, while adjacent WoS categories retained by platform indexing were evaluated in sensitivity analysis. Bibliometrix analyses comprised performance indicators, collaboration patterns, co-citation, keyword co-occurrence, thematic mapping and trend analysis on a harmonised keyword field.

**Results:**

The primary corpus comprised 271 documents from 97 sources, with 1311 authors and 16.3 mean citations per document. Output rose from low single digits before 2010 to 25–29 documents per year in 2022–2023; an exploratory logistic model indicated continued rapid growth. The Journal of Orthopaedic Trauma and Injury dominated the source landscape, while Germany, China, Switzerland and the United States led production. Foundational percutaneous-fixation studies and the subsequent navigation literature structured the intellectual base. Navigation, fluoroscopy and screw insertion formed the conceptual backbone, whereas explicit AI terminology appeared in only 1 of 271 documents at the author-keyword level.

**Conclusions:**

Percutaneous pelvic-ring fixation research is in sustained growth around a mature navigation backbone, while robotics, augmented reality and AI form a thin but identifiable emerging frontier. The findings support research prioritisation, cross-border collaboration and transparent reporting of new technologies in trauma orthopaedics.

**Supplementary Information:**

The online version contains supplementary material available at https://doi.org/10.1007/s00402-026-06562-3.

## Introduction

Pelvic ring injuries range from low-energy fragility fractures in older adults to high-energy disruptions associated with major haemorrhage and high mortality [1]. Restoration of mechanical stability is central to their management, and over the last three decades the operative treatment of the posterior pelvic ring has shifted decisively from open reduction towards minimally invasive, percutaneous techniques [2, 3]. Percutaneous iliosacral and trans-sacral screw fixation, LC-II (supra-acetabular) corridor screws and percutaneous rami screws now form the operative backbone for a large proportion of these injuries [3, 4].

The defining technical problem of percutaneous pelvic fixation is the placement of implants within narrow, three-dimensionally complex osseous corridors surrounded by neurovascular structures, which offer little margin for error [5]. Conventional fluoroscopy exposes both patient and surgical team to substantial ionising radiation and is limited by the difficulty of interpreting two-dimensional projections of a curved corridor. These limitations have driven successive waves of technological assistance: fluoroscopy-based and computed-tomography-based navigation [6, 7], intra-operative three-dimensional imaging, and, most recently, robotic guidance [8], augmented reality and artificial intelligence (AI). A recurring clinical question is whether these technologies measurably improve the accuracy and safety of screw placement and reduce radiation exposure, and how the supporting evidence has developed [9].

Bibliometric analysis provides a transparent, reproducible way to quantify the performance of a research field and to characterise its intellectual and conceptual structure through publication, citation and collaboration patterns [10, 11]. Science mapping extends classical bibliometrics by modelling networks of co-authorship, co-citation and keyword co-occurrence to identify thematic clusters, their maturity and their connectivity to the broader field [12, 13].

Bibliometric analyses of robot-assisted orthopaedic surgery have appeared recently, but none addresses percutaneous pelvic-ring fixation specifically. Guo et al. mapped orthopaedic surgical robotics as a whole [14], Deng et al. examined traumatic orthopaedic surgery robots over a single decade [15], and Yang et al. analysed robot-assisted orthopaedic surgery between 2005 and 2024 [16]. All three treated robotics as the unit of analysis across the entire specialty, so that the pelvic ring appears only as a minor sub-theme. None covered the full technological sequence from fluoroscopy through navigation to robotics and AI, none extended back to the codification of the percutaneous technique in the mid-1990s, and none reconstructed the citational base of a pelvis-specific literature through co-citation and reference-publication-year spectroscopy. A preliminary literature search did not identify a dedicated bibliometric and science-mapping analysis focused specifically on navigation, robotics and AI in percutaneous pelvic-ring fixation.

The present study mapped approximately three decades (1996–2026) of research on navigation, robotics and AI in percutaneous pelvic-ring fixation, using the Web of Science (WoS) Core Collection as the primary dataset and Scopus as a robustness comparison. The objectives were to: describe publication trends, growth dynamics and the life-cycle stage of the field; characterise its social structure through leading journals, countries, institutions, authors and collaboration patterns; reconstruct its intellectual structure and historical citation base; and map its conceptual structure and thematic evolution, with explicit attention to the emerging robotic and AI frontier.

## Materials and methods

### Databases and search strategy

Two databases were queried. The WoS Core Collection served as the primary dataset because, unlike the Scopus and merged exports, it provided fully parsed cited-reference metadata, a prerequisite for co-citation analysis, historiographic reconstruction and reference-publication-year spectroscopy [11, 17]. Scopus was used as a robustness comparison for the source and country landscapes, and a deduplicated WoS and Scopus corpus was examined as a coverage check; coverage is known to differ across the two platforms [18]. Both databases were interrogated on the same day, 8 June 2026, with identical conceptual logic and adapted syntax. A Topic or TITLE-ABS-KEY strategy combined a pelvic-fixation-technique block (iliosacral, sacroiliac, trans-sacral, trans-iliac, percutaneous pelvic, pelvic ring fixation or injury, posterior pelvic ring, LC2 corridor, pubic rami or ramus and anterior-column screws, sacropelvic fixation) with a technology block (artificial intelligence, machine or deep learning, neural networks, navigation, computer-assisted or computer-navigated surgery, robotics, augmented reality, 3D fluoroscopy, intra-operative imaging) and an exclusion block suppressing degenerative-spine, deformity and arthroplasty or acetabular noise. The verbatim WoS and Scopus strings and the stop-term list are reported in full in Online Resource 1 so that the search is transparent and reproducible [19].

### Document selection and corpus definition

**Fig. 1:**
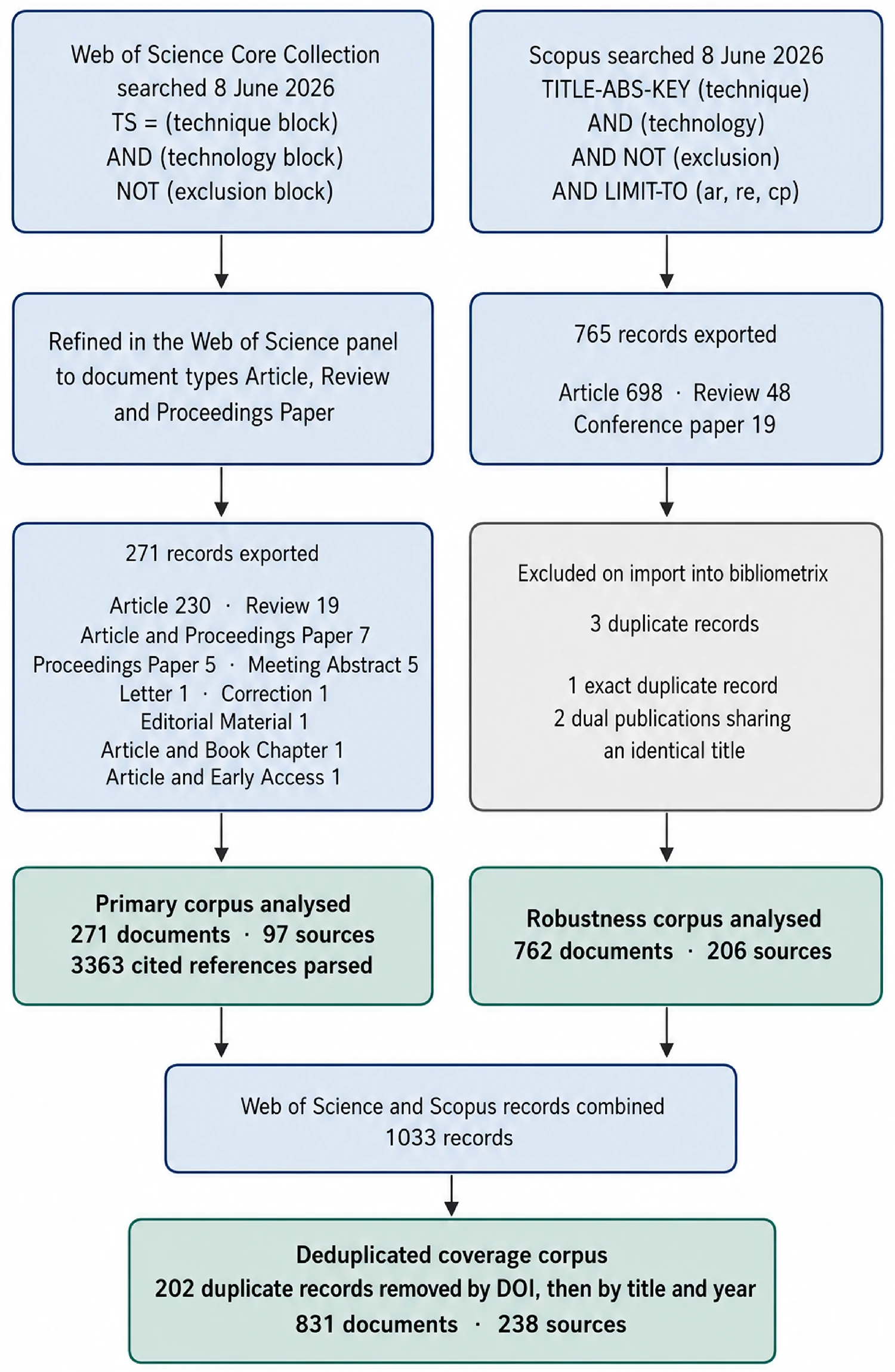
Selection and deduplication of records for the primary Web of Science corpus, the Scopus robustness corpus and the merged coverage corpus. Counts are those of the exported record sets on the search date of 8 June 2026. The document categories listed for Web of Science are the verbatim index categories of the exported records

Results were limited to articles, reviews and conference papers. Document types were restricted in the WoS refine panel to Article, Review and Proceedings Paper, and in Scopus through the LIMIT-TO operator for articles, reviews and conference papers. The exported WoS set retained eight records indexed under adjacent categories (five meeting abstracts, one letter, one correction and one editorial material), amounting to 2.9% of the corpus; these were retained so that the analysed corpus corresponds exactly to the exported record set, and their influence on the results was quantified in a sensitivity analysis reported under Limitations. Three duplicate records, one exact duplicate and two dual publications sharing an identical title, were removed from the Scopus export on import. For the coverage corpus, WoS and Scopus records were merged and deduplicated by DOI and then by title and year. The complete selection and deduplication sequence, with record counts for both databases and for the merged corpus, is shown in Fig. 1.

This study used bibliometric science-mapping methods rather than clinical effect synthesis. The full PRISMA checklist, which presupposes study-level eligibility screening, effect-size synthesis and risk-of-bias appraisal, was therefore not applicable to this design; the selection and deduplication sequence is nonetheless reported as a flow diagram (Fig. 1), and the verbatim search strategies in Online Resource 1 allow the corpus to be re-derived.

### Data collection and analytical workflow

Full-record and cited-reference files were exported from WoS, and full bibliographic records from Scopus. Retained fields included author and affiliation data, source and document titles, abstracts, publication years, document types, DOIs, author keywords, Keywords Plus or indexed keywords, citation counts and cited references where available. Records were imported into R and analysed with the bibliometrix package and its biblioshiny interface [11]. Performance analysis quantified annual scientific production, leading sources (with Bradford zoning), source impact (h-, g- and m-indices) [20, 21], leading countries (total counting and corresponding-author single- versus multi-country publications), most productive authors and affiliations, and the most globally and locally cited documents. Social-structure indicators comprised author and affiliation productivity together with corresponding-author country output and international collaboration patterns. Author productivity was assessed against Lotka’s law, and the growth trajectory was fitted with a logistic life-cycle model to estimate the inflection year and projected saturation level [22]. For readability, ranked figures display the leading 12 sources, six corresponding-author countries and 12 globally or locally cited documents; corpus-level indicators were calculated on the complete cleaned WoS set. Science mapping comprised keyword co-occurrence analysis, Callon centrality–density thematic mapping [12, 13], a multiple-correspondence factorial analysis, bibliographic-coupling clustering, trend-topic analysis, and, on the WoS corpus only, co-citation, historiographic and reference-publication-year spectroscopy analyses.

### Term harmonisation and stop-terms

A deliberate, prespecified cleaning step avoided spurious findings driven by database index terms. In the merged and Scopus exports the most frequent words were dominated by generic Emtree- or MeSH-style descriptors and publication-type tags carrying no topical meaning, for example human, male, female, article and priority journal; these were treated as stop-terms and removed before keyword co-occurrence, thematic labelling, factorial analysis and trend reporting. The full stop-term list is given in Online Resource 1. The substantive technique and technology terms, such as navigation, iliosacral screw and fluoroscopy, which constitute the actual topical content of the field, were retained. Because author keywords alone were sparse in this corpus, the conceptual-structure analyses and figures were built on the combined author-keyword and Keywords-Plus field after stop-term removal; reported keyword frequencies therefore include indexer-assigned Keywords-Plus terms as well as author keywords. Minor orthographic variants were unified, for example iliosacral and sacroiliac screw.

### Interpretive caution

Bibliometric indicators are descriptive signals of attention and diffusion, not measures of methodological quality [10]. Citation-based indicators are time-dependent and sensitive to the citation window, so the most recent years, in particular the partial year 2026, contribute volume but have had little time to accumulate citations and are interpreted accordingly. Absolute counts are reported descriptively within each platform, with cross-database concordance used to assess robustness [18].

### Software and use of AI tools

All analyses were performed in R with bibliometrix and biblioshiny [11]. Figures were prepared with matplotlib. AI tools (Claude, Anthropic, San Francisco, CA, USA) were used to proof, edit and enhance text; the authors take full responsibility for the content, interpretation and conclusions of the manuscript.

## Results

### Main information and corpus comparison

The primary WoS corpus comprised 271 documents from 97 sources (1996–2026), with 1311 authors, only 3 single-authored documents, 6.5 co-authors per document and 14.0% international co-authorship as shown in Table 1; the mean citation count was 16.3 per document. Of these records, 230 were indexed as Article and 19 as Review, 12 as Proceedings Paper or as Article and Proceedings Paper, and the remaining 10 under adjacent document categories (Fig. 1). For robustness, the Scopus corpus contained 762 documents (206 sources) and the deduplicated WoS and Scopus corpus 831 documents (238 sources); the larger counts reflect broader indexing rather than a different field [18]. Collaboration metrics were stable across the single-database corpora (international co-authorship 14.0% in WoS, 11.7% in Scopus) but fell implausibly to 4.6% in the merged corpus, an affiliation-parsing artefact on merge that reinforces the decision to derive collaboration and reference-based indicators from WoS.

**Table 1:** Main information for the primary Web of Science corpus and for the Scopus and merged corpora used for robustness

Indicator	WoS (primary)	Scopus	WoS + Scopus
Timespan	1996–2026	1994–2026	1994–2026
Documents	271	762	831
Sources	97	206	238
Annual growth rate (%)	7.6	11.4	12.1
Mean citations per document	16.3	20.3	18.4
Authors	1311	2811	2998
Co-authors per document	6.5	5.6	5.7
International co-authorship (%)	14.0	11.7	4.6 (artefact)
Cited references parsed	3363	0	0
*Document categories*			
Article	230	698	–
Review	19	48	–
Proceedings or conference paper	12	19	–
Other categories	10	0	–

*WoS* Web of Science

Cited references were parsed only in Web of Science. Document categories are the verbatim index categories of the exported record sets; the merged corpus was not re-classified by document category.

### Annual production and life-cycle

**Fig. 2:**
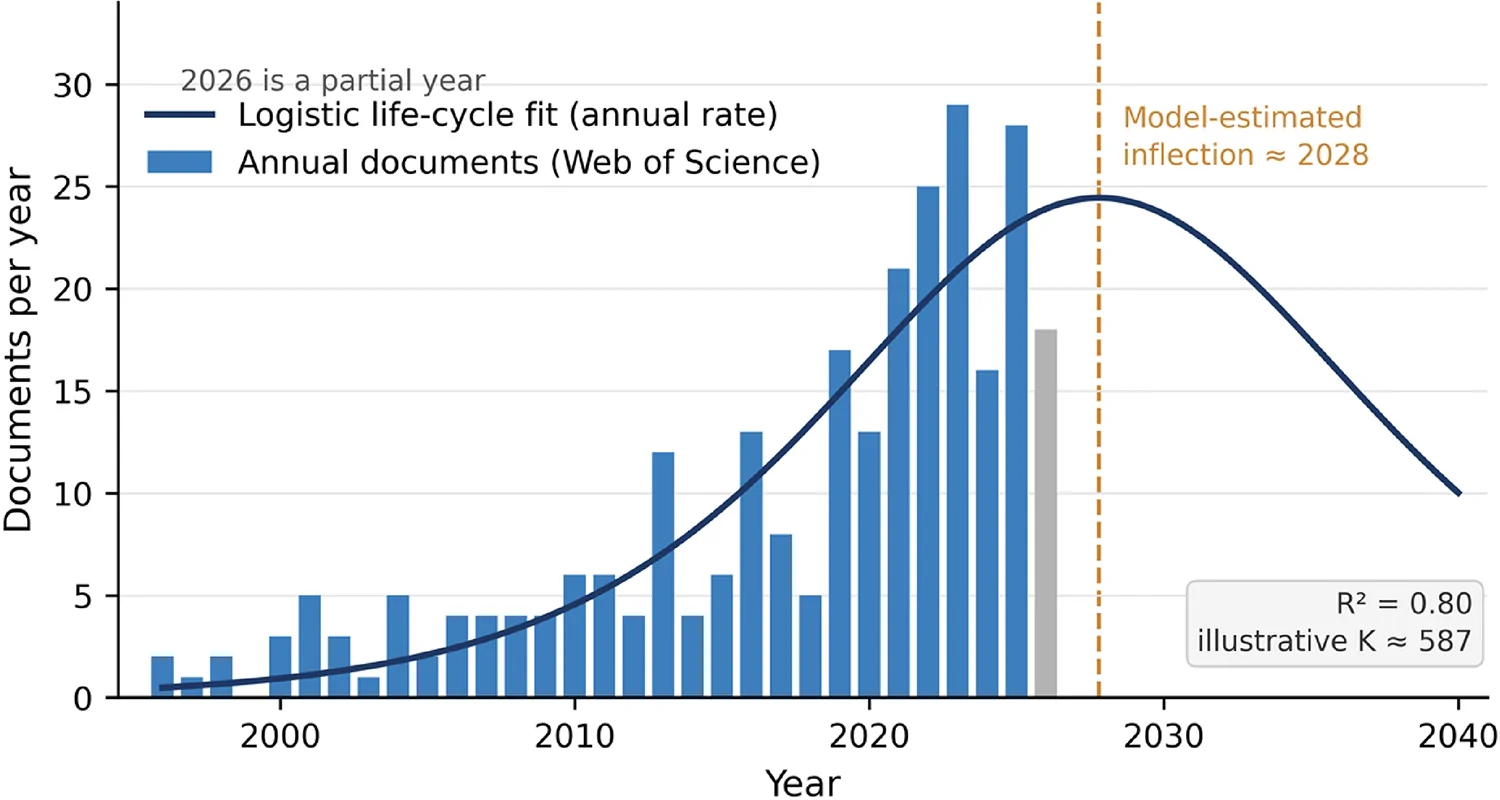
Observed annual scientific production and exploratory logistic life-cycle fit in the Web of Science corpus. Bars represent annual document counts and the grey bar represents the partial year 2026. The solid line represents the first derivative of the fitted cumulative logistic and reaches its model-estimated maximum near 2028, marked by the vertical dashed line. The estimated saturation parameter was model-dependent and is presented descriptively rather than as a forecast

Annual output was low and irregular before 2010 (typically 1–6 documents per year), reached 12–13 documents in 2013 and 2016, and accelerated after 2018, with 17 documents in 2019, 21 in 2021, 25 in 2022 and a peak of 29 in 2023 (28 in 2025; 2026 a partial year with 18 records to the search date). An exploratory logistic life-cycle model was fitted to the annual output (R² = 0.80). Figure 2 displays the observed annual counts together with the fitted annual rate, the first derivative of the cumulative logistic, which rises, peaks at the model-estimated inflection (≈ 2028) and then declines as cumulative output approaches saturation. On this model the field is still in its rapid-growth phase, below half of the projected cumulative total, with the inflection not yet reached. The saturation level is, however, highly sensitive to model specification and to inclusion of the partial final year, and is reported only as an illustrative model parameter (K ≈ 587 cumulative documents) rather than a firm forecast; the robust, specification-independent conclusion is simply that output remains in accelerating growth, so that substantial further output is expected over the coming years.

### Leading sources and Bradford core

**Fig. 3:**
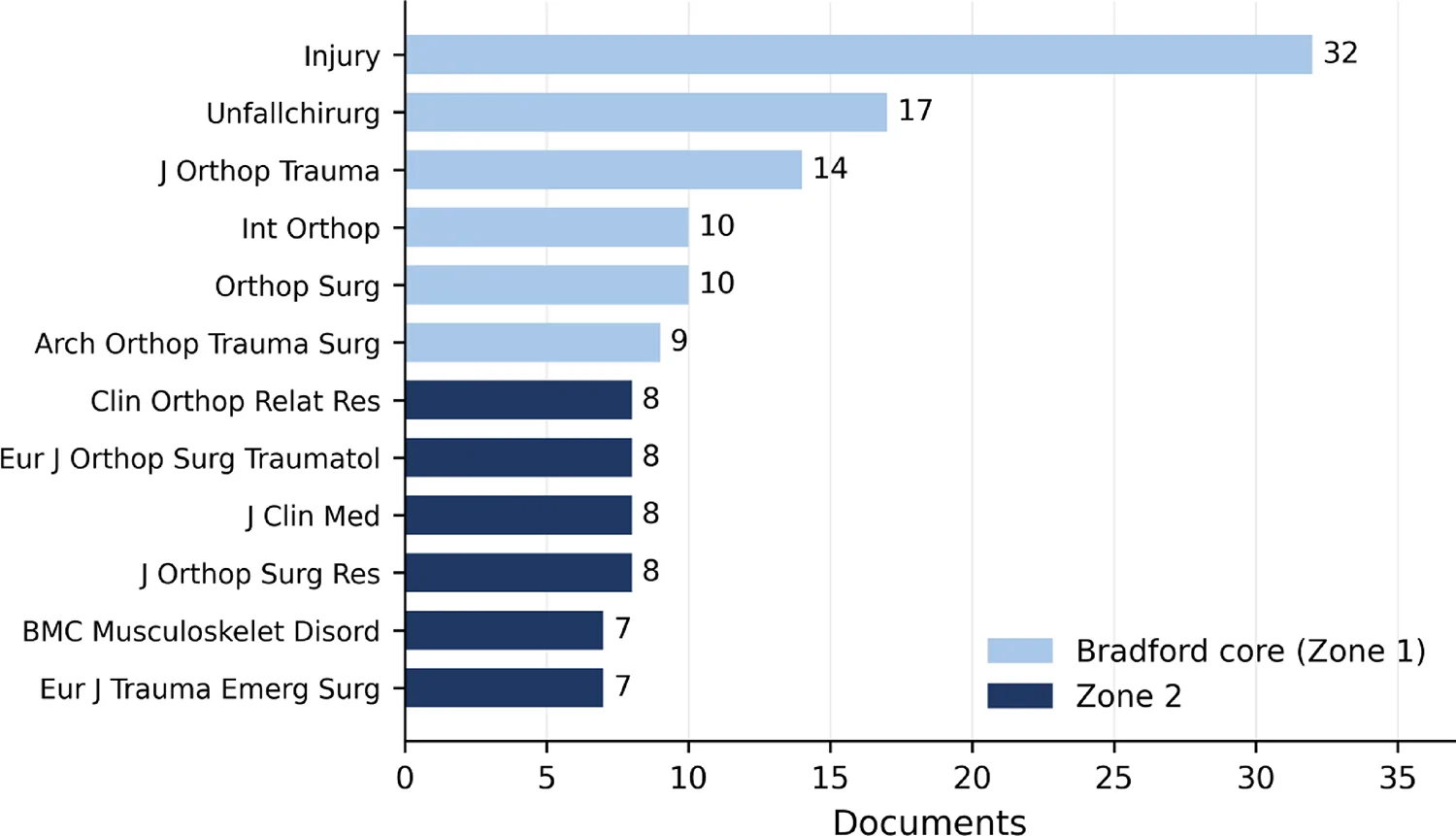
Most productive sources in the primary Web of Science corpus. Light bars identify the six journals belonging to the Bradford core (Zone 1) and dark bars identify journals in Zone 2. Indexing variants of Injury were harmonised before source counts were calculated. Journal titles are given as standard abbreviations

Publication output followed a classic Bradford distribution as shown in Fig. 3. In WoS the core zone comprised six journals, Injury (32 documents), Der Unfallchirurg (17), Journal of Orthopaedic Trauma (14), International Orthopaedics (10), Orthopaedic Surgery (10) and Archives of Orthopaedic and Trauma Surgery (9); the Journal of Robotic Surgery appeared in the second Bradford zone, consistent with robotics as an emerging peripheral theme. Across databases the two dominant venues were Injury and the Journal of Orthopaedic Trauma: in Scopus the order reversed (Journal of Orthopaedic Trauma 73, Injury 65) and in the merged corpus the Journal of Orthopaedic Trauma led (75) with Injury close behind once its two indexing variants were combined (≈ 74). By source impact the Journal of Orthopaedic Trauma was the strongest single journal (h = 29, g = 58, m = 0.9, 3421 citations) and the single most-cited source in the reference base (993 citing references), ahead of Injury (696) and Clinical Orthopaedics and Related Research (598). Injury, by contrast, was the most productive current venue in WoS, whereas the Journal of Orthopaedic Trauma represented the most-cited knowledge base.

### Countries and collaboration

**Fig. 4:**
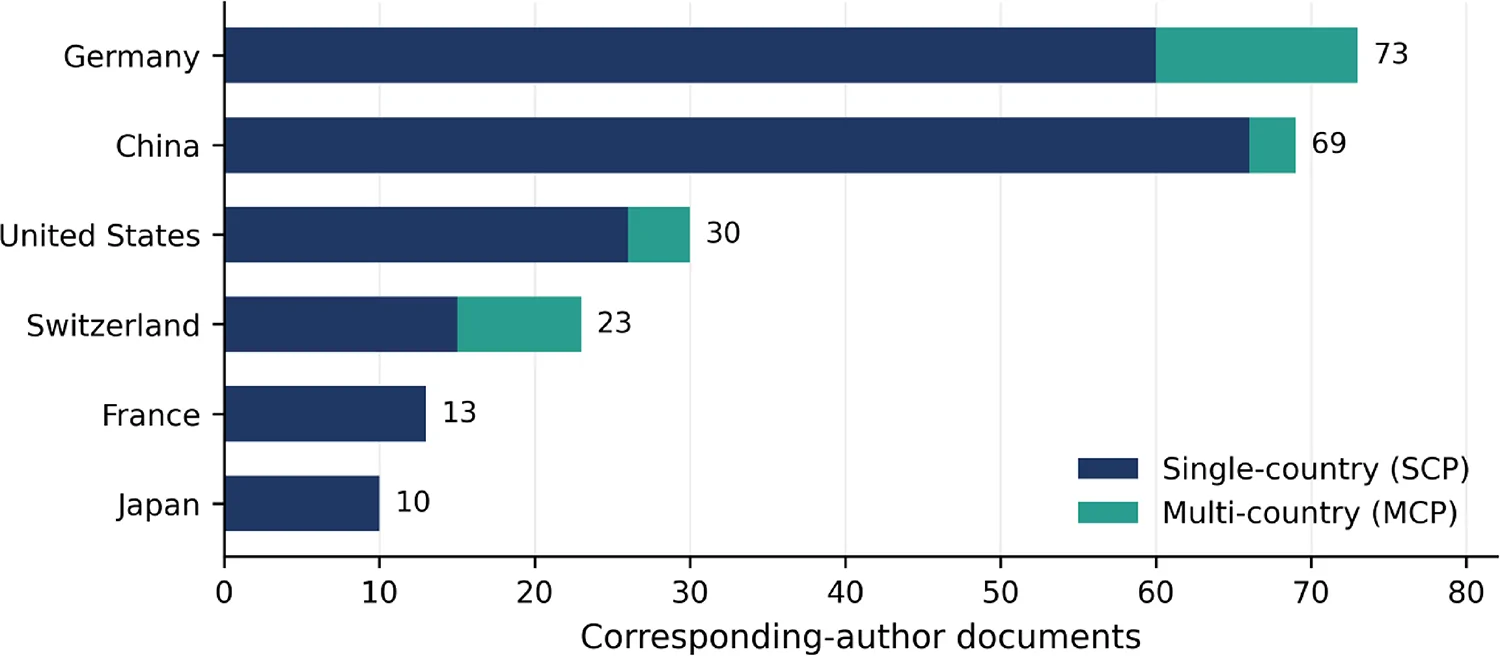
Leading countries by corresponding-author output in the Web of Science corpus. Bars are divided into single-country publications (SCP) and multi-country publications (MCP), and labels indicate total corresponding-author documents

Corresponding-author analysis identified Germany (73 documents; 26.9%), China (69; 25.5%), the United States (30; 11.1%) and Switzerland (23; 8.5%) as the leading producing countries as shown in Fig. 4; Table 2; the same four led across databases despite coverage-driven order changes. The citation profile was distinctly geographical: Germany accumulated by far the most citations (1848; mean 25.3 per article), reflecting the German-speaking origins of computer-assisted orthopaedic surgery. International collaboration was highly uneven: multi-country publication rates were high for Switzerland (34.8%) and Austria (40%) but very low for China (4.3%), indicating a largely domestic Chinese output and an opportunity for cross-border consortia, particularly relevant for navigation and AI applications that are sensitive to population, equipment and workflow context.

**Table 2:** Leading countries by corresponding-author output, multi-country publication share and citation impact (Web of Science)

Country	Corr.-author docs	%	MCP (%)	Total citations	Mean cit./art.
Germany	73	26.9	17.8	1848	25.3
China	69	25.5	4.3	872	12.6
United States	30	11.1	13.3	506	16.9
Switzerland	23	8.5	34.8	252	11.0
France	13	4.8	0	157	12.1
Japan	10	3.7	0	129	12.9

*Corr.-author docs* corresponding-author documents,* MCP* multi-country publication,* cit./art.* citations per article

### Authors and institutions

Author productivity followed Lotka’s law, with 80.6% of authors contributing a single document and a maximum of eleven documents by the most prolific author (F. Gebhard); other recurrent contributors included C. Krettek and L.-P. Nolte, names closely associated with the development of trauma navigation [6]. The most productive institutions were the University of Zurich (20 documents), Hebei Medical University (19), the University of Bern (16), Gachon University (15), and the University of British Columbia and the University of Hamburg (13 each), underscoring the Swiss and German cradle of computer-assisted orthopaedic surgery alongside high-volume Chinese and Korean centres.

### Intellectual structure: cited documents, co-citation and historical references

**Fig. 5:**
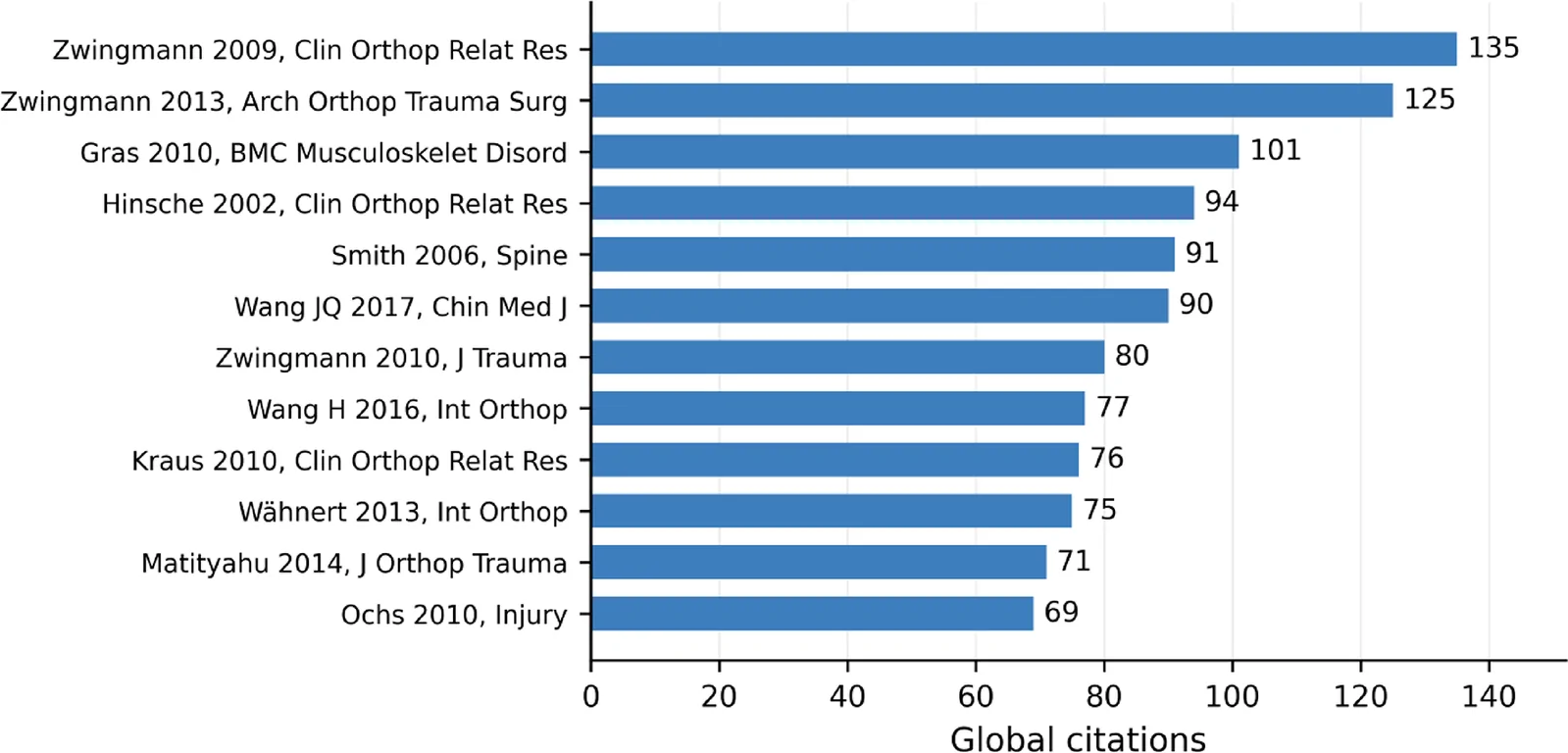
Most globally cited documents in the primary Web of Science corpus. Labels report first author, publication year and abbreviated journal title, and values represent total global citations recorded at the search date

The most globally cited documents of the primary corpus were the navigation-outcome studies of Zwingmann (2009, 135 citations [9]; 2013, 125 [23]; 2010, 80 [24]) and Gras (2010, 101 [25]), together with Hinsche (2002, 94 [26]), Smith (2006, 91 [27]) and the robot-assisted series of Wang JQ (2017, 90 [8]); other frequently cited works included Wang H (2016) [28], Kraus (2010) [29], Wähnert (2013) [30], Matityahu (2014) [31] and Ochs (2010) [32] (Fig. 5).

**Fig. 6:**
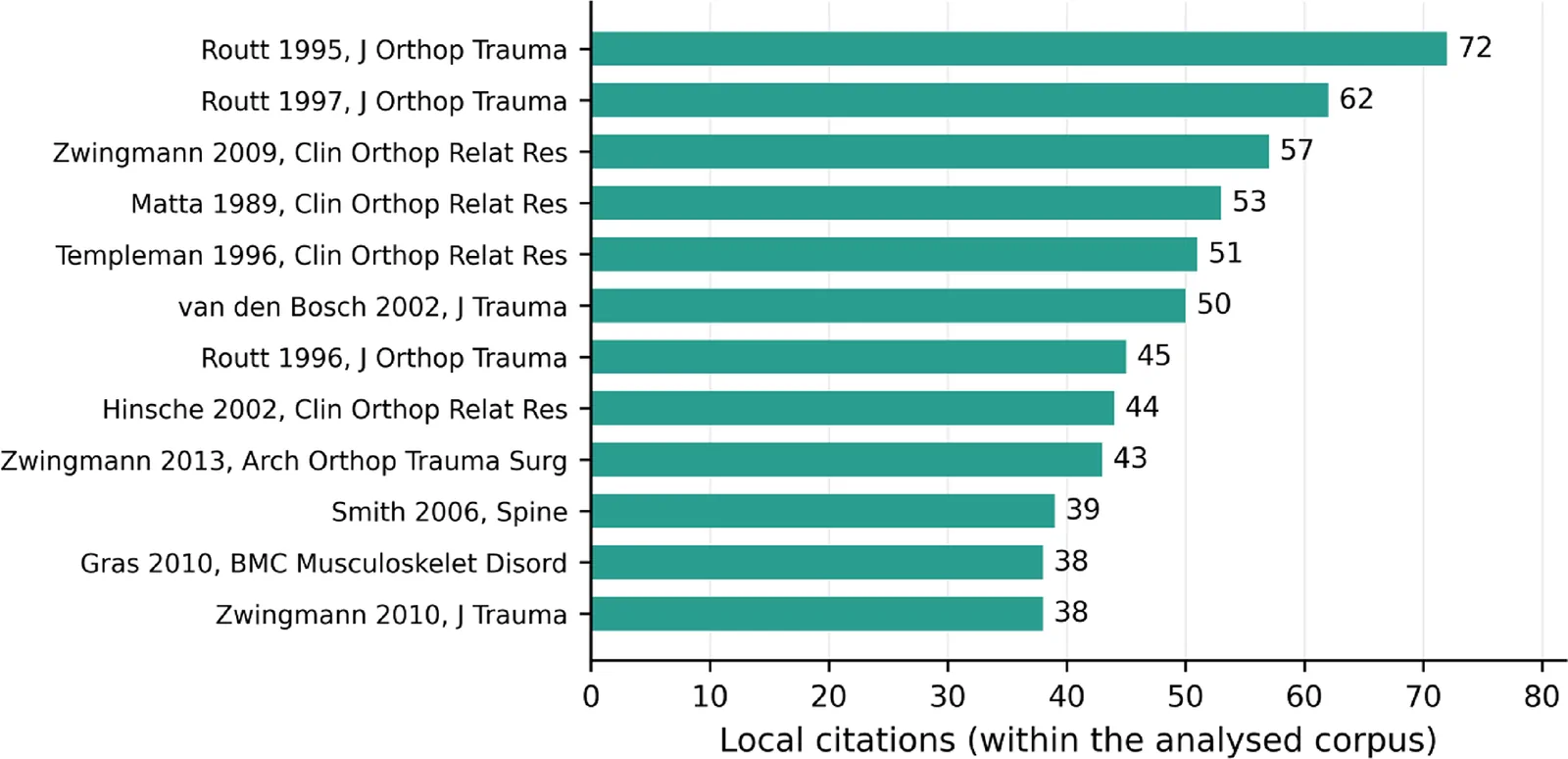
Most locally cited references in the primary Web of Science corpus. Local citations represent citations received from documents included within the analysed corpus. Labels report first author, publication year and abbreviated journal title

The locally most-cited references, which define the intellectual base of the corpus, were the foundational percutaneous-fixation papers of Routt (1995 [2]; 1997 [3]; 1996 [4]), the Zwingmann navigation study [9], and the classic internal-fixation and outcome studies of Matta (1989) [33], Templeman (1996) [34] and van den Bosch (2002) [35], with further anchors in Ertel (2001) [1], Bellabarba (2006) [36], Sagi (2011) [37] and Gardner (2010–2011) [38, 39]. The complete ranking of the most locally cited references is shown in Fig. 6.

Co-citation, local-citation and historical-reference analyses converged on the same intellectual structure. Routt’s studies formed the foundational axis of percutaneous iliosacral fixation, while Zwingmann and Gras defined the principal navigation-outcome lineage.

Reference-publication-year spectroscopy revealed the historical scaffolding of the field as a sequence of citation peaks: a 1980 cluster of classification work (Tile [40], Pennal [41], Letournel [42]), a 1989 cluster on internal fixation and outcome scoring (Matta [33], Majeed [43]), and a dominant 1995–1996 peak marking the codification of the percutaneous technique (Routt [2, 4], Templeman [34]), followed by a sustained 2000–2013 plateau corresponding to the navigation era (Carlson [44], Amiot [7], Stöckle [45], Konrad [46], König [47], Gras [25], Zwingmann [23]). Image-guided-surgery and navigation references (Nolte [6], Schwarzenbach [48], Takao [5], Wong [49]) and corridor-morphometry work (Mendel [50]) anchor, respectively, the technological lineage and the safe-corridor literature; the durable influence of the original biomechanical question is reflected in Tile (1988) [51] and Ochs (2010) [32].

### Conceptual structure and thematic evolution

**Fig. 7:**
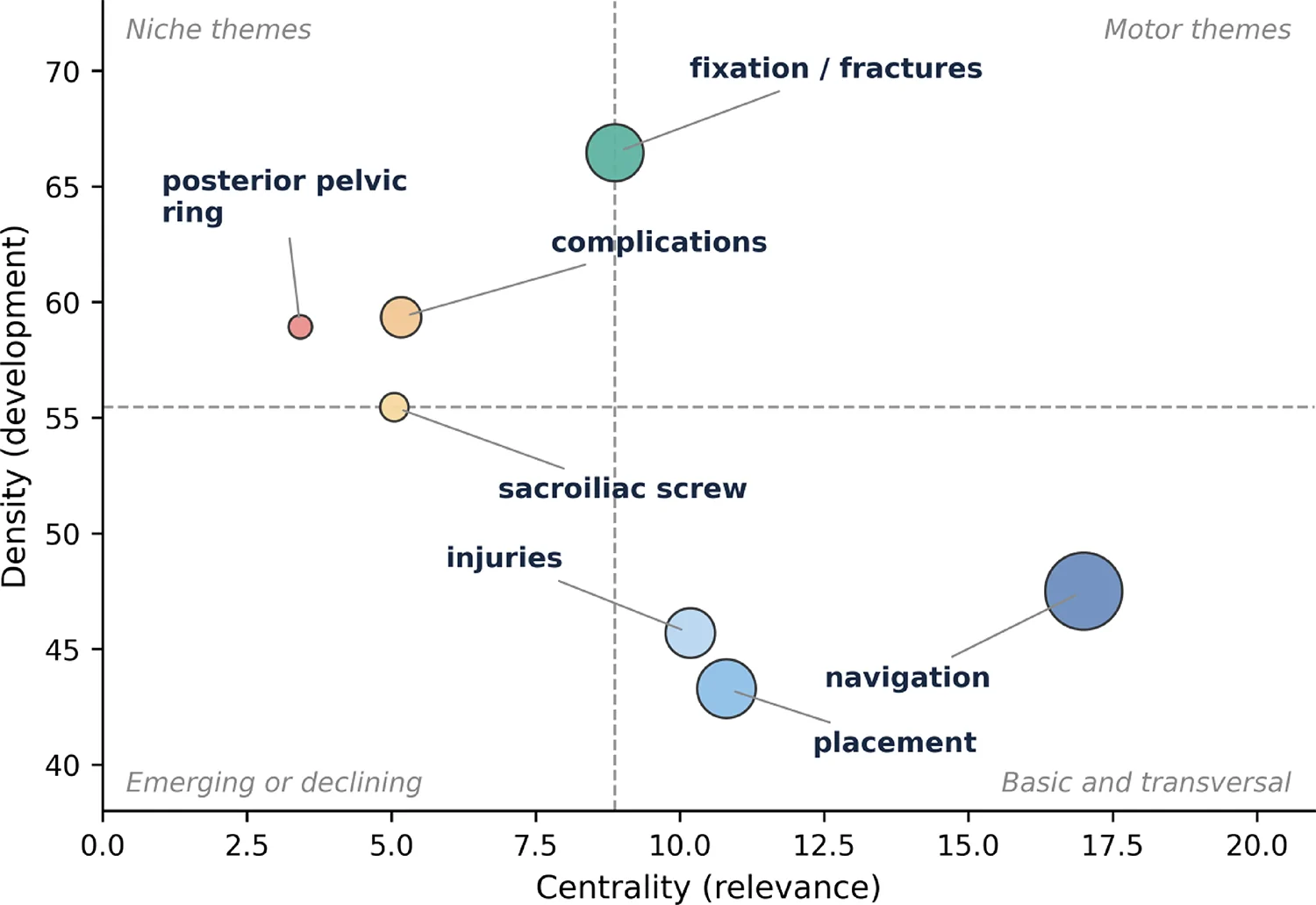
Callon thematic map based on the combined Web of Science author-keyword and Keywords-Plus field after term harmonisation and stop-term removal. Centrality represents thematic relevance and density represents internal development, bubble size is proportional to cluster frequency, and dashed lines mark the median centrality and density. Navigation occupied the central basic and transversal quadrant, whereas fixation and fractures was the most developed cluster and lay near the motor-theme boundary

After stop-term removal from the combined author-keyword and Keywords-Plus field (Online Resource 1), the leading topical terms were navigation (99), fixation (91), placement (85), fluoroscopy (58), insertion (57), iliosacral screws (54), accuracy (38), internal fixation (38) and complications (32). Callon thematic mapping (Fig. 7) positioned surgical navigation, fluoroscopy and screw insertion as a large, highly central but only moderately developed cluster, the transversal, basic backbone of the field, alongside placement, internal fixation and iliosacral screw, and injuries, pelvis and reduction. Fracture-fixation outcomes formed the most internally developed (motor) theme, while complications, the posterior pelvic ring and sacroiliac-screw technique occupied specialised niche positions (Table 3).

**Table 3:** Thematic clusters with Callon centrality and density (Web of Science author keywords and Keywords-Plus)

Cluster (label)	Centrality	Density	Frequency	Position
Navigation	17.0	47.5	696	central backbone
Placement	10.8	43.3	403	basic/transversal
Injuries	10.2	45.7	288	basic
Fixation/fractures	8.9	66.5	380	borderline motor/highly developed
Complications	5.2	59.3	189	niche
Sacroiliac screw	5.1	55.5	94	niche
Posterior pelvic ring	3.4	58.9	64	niche

Centrality and density are Callon co-word indices; frequency is the term count in the combined keyword field

### Factorial structure, bibliographic coupling and emerging trends

**Fig. 8:**
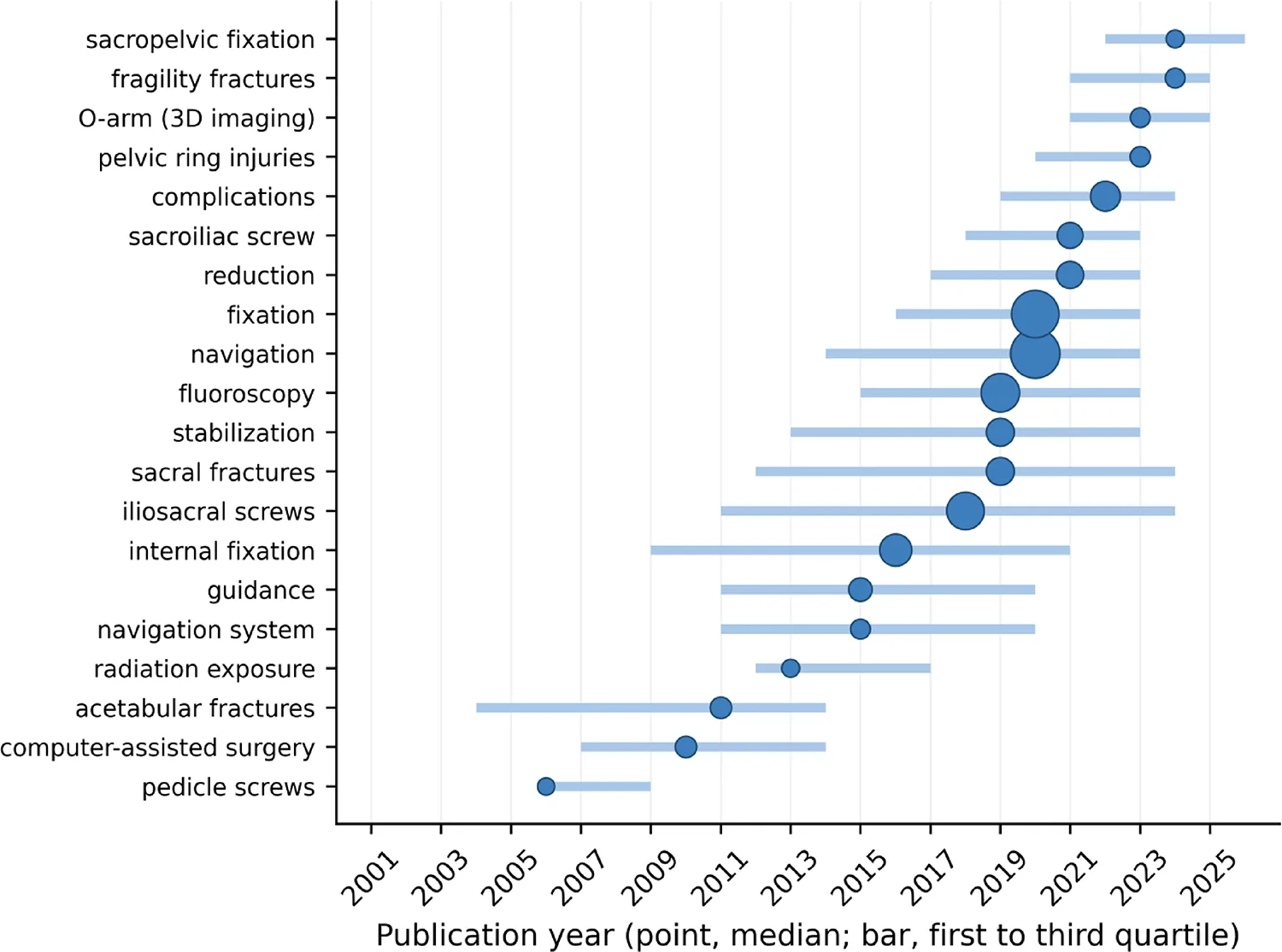
Temporal distribution of trend topics in the Web of Science corpus after term harmonisation and stop-term removal. Each point is positioned at the median publication year, horizontal bars represent the interquartile range, and point size is proportional to term frequency. Acetabular fractures was an indexer-assigned Keywords-Plus term associated with retained mixed pelvic-ring or corridor-screw records and did not represent isolated acetabular reconstruction

Trend-topic analysis (Fig. 8) traced a clear technological evolution: an early phase of computer-assisted surgery and pedicle-screw work (median year ≈ 2002–2010), the latter reflecting early lumbopelvic and triangular-fixation constructs rather than spinal-deformity surgery; a middle phase of CT-based and fluoroscopic navigation, navigation systems, image guidance and radiation-exposure concerns (≈ 2013–2016); and a recent phase dominated by iliosacral-screw placement, fluoroscopy, navigation, reduction and complications (≈ 2018–2021). The most recent emergent terms (median year ≥ 2023) were intra-operative 3D imaging (O-arm), fragility fractures of the pelvis and sacropelvic fixation

The multiple-correspondence factorial analysis separated a technology-facing pole (robotics, minimally invasive surgery and lumbopelvic fixation) from an injury-pattern pole, while bibliographic-coupling clustering identified augmented and mixed reality as low-centrality emerging terms. Explicit AI terminology was rare: artificial-intelligence, machine-learning or deep-learning terms appeared in only 1 of 271 documents (0.4%) at the author-keyword level and 5 (1.8%) across titles, abstracts and keywords, against 114 documents (42%) carrying navigation or computer-assisted-surgery terminology and 40 (15%) carrying robotics; AI in this domain is therefore at an early, pre-diffusion stage rather than an established theme. The Keywords-Plus term acetabular fractures visible in Fig. 8 denotes retained mixed pelvic-ring or corridor-screw records co-indexed under that plural form, not isolated acetabular reconstruction.

### Cross-database robustness

The leading-source and leading-country landscapes were directionally consistent across WoS, Scopus and the merged corpus: the Journal of Orthopaedic Trauma and Injury co-led the journals in every database, and the United States, China, Germany and Switzerland led the countries throughout, with rank changes attributable to known coverage differences, as Scopus indexes the Journal of Orthopaedic Trauma and US output more heavily [18]. This convergence supports interpreting the WoS-derived structure as generalisable rather than database-specific.

## Discussion

This bibliometric and science-mapping analysis of percutaneous pelvic-ring fixation describes a field in sustained, accelerating growth, organised around a stable conceptual backbone, with a thin but identifiable technological frontier. Three findings stand out: a navigation-centred conceptual core; a distinctive geography in which a highly collaborative German and Swiss tradition coexists with a large, largely domestic Chinese contribution; and a late-period emergence of intra-operative 3D imaging, fragility-fracture management, robotics and augmented reality, with AI still at a pre-diffusion stage.

### Intellectual and conceptual structure of the field

The intellectual and conceptual structures were mutually coherent and clinically plausible. Co-citation and reference spectroscopy identified the percutaneous-fixation lineage on which the technology literature developed, while surgical navigation, fluoroscopy and screw insertion formed a central, transversal backbone because the core technical problem—accurate placement of screws in narrow sacral and supra-acetabular corridors with minimal radiation [5, 9]—cuts across every sub-topic. Fixation outcomes constituted the most developed theme, while complications, the posterior pelvic ring and sacroiliac-screw technique remained specialised niches. The historical scaffolding extended from the 1980 classifications [40–42] and 1989 internal-fixation literature [33, 43] to the 1995–1996 codification of the percutaneous technique by Routt [2, 4], showing that successive technological waves were layered onto a durable practice-defining base rather than replacing it.

### Geography of production and collaboration

The geography of the field is informative for collaboration policy. Germany and Switzerland produced the most-cited work and collaborated internationally at high rates, consistent with the German-speaking origins of computer-assisted orthopaedic surgery and the prominence of Bern and Zurich [6]. The output of China has grown rapidly but with very low multi-country collaboration, suggesting an opportunity for cross-border consortia, particularly relevant for navigation and AI applications, whose performance is sensitive to population, equipment and workflow context and therefore benefits from external validation [18].

### Research trends and the emerging technological frontier

The emerging frontier deserves careful, honest framing. Navigation and computer-assisted surgery are the established, evidence-bearing core of the field [9, 25]; robotics [8] and augmented reality appear as genuine but still peripheral emerging terms, and explicit AI or machine-learning terminology is currently sparse in this specific domain. This distribution argues against overstating an AI revolution in percutaneous pelvic fixation: the accurate description is of a mature navigation paradigm onto which robotics, mixed reality and, prospectively, AI are beginning to be layered. The parallel late-period emergence of fragility fractures of the pelvis and sacropelvic fixation reflects the demographic shift towards an older, osteoporotic population and is likely to be a major driver of future output.

### Implications for research and reporting

From an editorial standpoint, the compact Bradford core and the rapid-growth life-cycle stage of the field favour method- and governance-focused contributions: reporting standards for navigation and robotic accuracy, radiation-dose transparency and prespecified validation of AI tools, concentrated in the leading trauma journals of the field. Across databases, the Journal of Orthopaedic Trauma emerged as the single most-cited source and the strongest journal by impact (h = 29, g = 58, m = 0.9), forming the citational knowledge base of the field, while Injury was the most productive current venue; together they constituted the two dominant trauma-orthopaedic outlets.

For the field to convert technological promise into demonstrable clinical benefit, future studies should converge on a consistent core of reported outcomes. These include the accuracy of screw placement against an explicit breach definition, the revision and hardware-failure rate, radiation dose to both patient and surgical team, navigation or robotic set-up and workflow time, the learning curve, and the incremental cost of the technology. Reports of AI- and robotic-guidance systems should additionally provide prospective external validation rather than single-centre development data, and outcomes should be reported separately for fragility fractures of the pelvis, whose osteoporotic bone and distinct demographic make them a likely major driver of future work. Standardising this reporting set would allow the navigation, robotic and AI literature to be compared and pooled, moving the field from feasibility description towards evidence of patient benefit.

## Limitations

Bibliometric indicators are descriptive proxies for attention and diffusion, not measures of quality, and are sensitive to database coverage, indexing practices and the citation window [10, 18]; the partial year 2026 under-represents recent output and citations. Keyword-based mapping depends on indexing completeness, which is why the combined author-keyword and Keywords-Plus field was cleaned of metadata noise before mapping (Online Resource 1). Source-name variants, notably the two indexing forms of Injury, and a parser artefact in the affiliation field required manual correction. Co-citation and reference-based analyses were available only for WoS.

A sensitivity analysis addressed the eight WoS records indexed under document categories adjacent to the declared restriction. Excluding them reduced the corpus from 271 to 263 documents and the source count from 97 to 94, changed no annual count by more than three documents, and left the six-journal Bradford core and its rank order unchanged; because meeting abstracts, letters and corrections carry no parsed cited references, the co-citation, reference-publication-year and historiographic analyses were unaffected. One of these records is the conference abstract of a systematic review whose journal version is also present in the corpus, so that work is represented twice, affecting the 2024 and 2025 counts by one document each.

The exclusion block used the singular quoted phrase acetabular fracture and was directed at arthroplasty and isolated acetabular reconstruction, so that supra-acetabular (LC-II) and anterior-column corridor work remained well represented in the retained corpus (18 documents) and only a few mixed pelvic-ring records co-indexed under the plural acetabular fractures were retained; some borderline papers indexed solely as acetabular fractures may nonetheless have been missed. Finally, the search strategy, though designed to be sensitive and reproducible, may have missed work indexed under divergent terminology; the verbatim strings (Online Resource 1) make this trade-off transparent and allow the corpus to be re-derived.

## Conclusion

Research on navigation, robotics and AI in percutaneous pelvic-ring fixation has grown steadily over three decades and remains in an accelerating, rapid-growth phase: an exploratory logistic model places its model-estimated inflection beyond the observed period (around 2028), with the inflection not yet reached; the projected saturation level is sensitive to model specification and is reported only as an illustrative parameter rather than a forecast. The field is structured around a stable navigation, fluoroscopy and screw-placement backbone, built on the foundational percutaneous-fixation literature, with a compact core of trauma-orthopaedic journals led by the Journal of Orthopaedic Trauma and Injury and a research base concentrated in German-speaking Europe, China and North America. Robotics, augmented reality and the management of fragility fractures of the pelvis represent the principal emerging directions, while AI remains an early, pre-diffusion frontier whose translation into the operating room should be coupled to transparent reporting and prospective external validation. These maps can support research prioritisation, collaboration policy and editorial planning in trauma orthopaedics.

## Supplementary Information

Below is the link to the electronic supplementary material.

Online Resource 1 Search strategy, verbatim search strings and stop-term list (PDF). Cited in Materials and methods (Databases and search strategy; Document selection and corpus definition; Term harmonisation and stop-terms)

## Data Availability

The bibliographic records analysed in this study were retrieved from the Web of Science Core Collection and Scopus. As these databases are proprietary and their record exports are subject to licensing restrictions, the raw bibliographic data cannot be deposited in a public repository. The complete search strategies, search date, eligibility criteria, selection and deduplication procedures, and stop-term list are provided in Online Resource 1. All derived data supporting the findings are reported in the article, tables, figures and supplementary material.

## References

[1] Ertel W, Keel M, Eid K, Platz A, Trentz O (2001) Control of severe hemorrhage using C-clamp and pelvic packing in multiply injured patients with pelvic ring disruption. J Orthop Trauma 15:468–474. 10.1097/00005131-200109000-00002 11602828

[2] Routt MLC, Kregor PJ, Simonian PT, Mayo KA (1995) Early results of percutaneous iliosacral screws placed with the patient in the supine position. J Orthop Trauma 9:207–214. 10.1097/00005131-199506000-00005 7623172

[3] Routt MLC, Simonian PT, Mills WJ (1997) Iliosacral screw fixation: early complications of the percutaneous technique. J Orthop Trauma 11:584–589. 10.1097/00005131-199711000-00007 9415865

[4] Routt MLC, Simonian PT, Agnew SG, Mann FA (1996) Radiographic recognition of the sacral alar slope for optimal placement of iliosacral screws: a cadaveric and clinical study. J Orthop Trauma 10:171–177. 10.1097/00005131-199604000-00005 8667109

[5] Takao M, Nishii T, Sakai T, Yoshikawa H, Sugano N (2014) Iliosacral screw insertion using CT-3D-fluoroscopy matching navigation. Injury 45:988–994. 10.1016/j.injury.2014.01.015 24507831

[6] Nolte LP, Visarius H, Arm E, Langlotz F, Schwarzenbach O, Zamorano L (1995) Computer-aided fixation of spinal implants. J Image Guid Surg 1(2):88–93. (**10.1002/(sici)1522-712x(1995)1:2<88::aid-igs3>3.0.co;2-h**) 10.1002/(SICI)1522-712X(1995)1:2<88::AID-IGS3>3.0.CO;2-H 9079432

[7] Amiot LP, Lang K, Putzier M, Zippel H, Labelle H (2000) Comparative results between conventional and computer-assisted pedicle screw installation in the thoracic, lumbar, and sacral spine. Spine 25:606–614. 10.1097/00007632-200003010-00012 10749638

[8] Wang JQ, Wang Y, Feng Y, Han W, Su YG, Liu WY et al (2017) Percutaneous sacroiliac screw placement: a prospective randomized comparison of robot-assisted navigation procedures with a conventional technique. Chin Med J 130:2527–2534. 10.4103/0366-6999.217080 PMC5678249 29067950

[9] Zwingmann J, Konrad G, Kotter E, Südkamp NP, Oberst M (2009) Computer-navigated iliosacral screw insertion reduces malposition rate and radiation exposure. Clin Orthop Relat Res 467:1833–1838. 10.1007/s11999-008-0632-6 PMC2690740 19034594

[10] Donthu N, Kumar S, Mukherjee D, Pandey N, Lim WM (2021) How to conduct a bibliometric analysis: an overview and guidelines. J Bus Res 133:285–296. 10.1016/j.jbusres.2021.04.070

[11] Aria M, Cuccurullo C (2017) Bibliometrix: an R-tool for comprehensive science mapping analysis. J Informetr 11:959–975. 10.1016/j.joi.2017.08.007

[12] Cobo MJ, López-Herrera AG, Herrera-Viedma E, Herrera F (2011) Science mapping software tools: review, analysis, and cooperative study among tools. J Am Soc Inf Sci Technol 62:1382–1402. 10.1002/asi.21525

[13] Callon M, Courtial JP, Laville F (1991) Co-word analysis as a tool for describing the network of interactions between basic and technological research. Scientometrics 22:155–205. 10.1007/bf02019280

[14] Guo X, Wang D, Li J, Wang Y, Wang W, Bai W et al (2023) Global research status and trends in orthopaedic surgical robotics: a bibliometric and visualisation analysis study. J Robot Surg 17:1743–1756. 10.1007/s11701-023-01579-x 37017859

[15] Deng X, Wen Y, Lin H, Lan J, Li L (2025) Research on traumatic orthopedic surgery robots: a decade bibliometric analysis of research landscapes and evolving frontiers. J Robot Surg 19:705. 10.1007/s11701-025-02875-4 41117953

[16] Yang Z, Chang X, Fu G, Wang Y, Zhang X, Wang Z et al (2026) Global research hotspots and emerging trends in orthopedic robotic surgery: a comprehensive bibliometric analysis. J Robot Surg 20:179. 10.1007/s11701-025-02998-8 PMC12808197 41540157

[17] van Eck NJ, Waltman L (2010) Software survey: VOSviewer, a computer program for bibliometric mapping. Scientometrics 84:523–538. 10.1007/s11192-009-0146-3 PMC2883932 20585380

[18] Mongeon P, Paul-Hus A (2016) The journal coverage of Web of Science and Scopus: a comparative analysis. Scientometrics 106:213–228. 10.1007/s11192-015-1765-5

[19] Gusenbauer M, Haddaway NR (2020) Which academic search systems are suitable for systematic reviews or meta-analyses? Evaluating retrieval qualities of Google Scholar, PubMed, and 26 other resources. Res Synth Methods 11:181–217. 10.1002/jrsm.1378 PMC7079055 31614060

[20] Hirsch JE (2005) An index to quantify an individual’s scientific research output. Proc Natl Acad Sci U S A 102:16569–16572. 10.1073/pnas.0507655102 PMC1283832 16275915

[21] Egghe L (2006) Theory and practise of the g-index. Scientometrics 69:131–152. 10.1007/s11192-006-0144-7

[22] Zupic I, Čater T (2015) Bibliometric methods in management and organization. Organ Res Methods 18:429–472. 10.1177/1094428114562629

[23] Zwingmann J, Hauschild O, Bode G, Südkamp NP, Schmal H (2013) Malposition and revision rates of different imaging modalities for percutaneous iliosacral screw fixation following pelvic fractures: a systematic review and meta-analysis. Arch Orthop Trauma Surg 133:1257–1265. 10.1007/s00402-013-1788-4 23748798

[24] Zwingmann J, Konrad G, Mehlhorn AT, Südkamp NP, Oberst M (2010) Percutaneous iliosacral screw insertion: malpositioning and revision rate of screws with regards to application technique (navigated vs. conventional). J Trauma 69:1501–1506. 10.1097/ta.0b013e3181d862db 20526214

[25] Gras F, Marintschev I, Wilharm A, Klos K, Mückley T, Hofmann GO (2010) 2D-fluoroscopic navigated percutaneous screw fixation of pelvic ring injuries: a case series. BMC Musculoskelet Disord 11:153. 10.1186/1471-2474-11-153 PMC2916892 20609243

[26] Hinsche AF, Giannoudis PV, Smith RM (2002) Fluoroscopy-based multiplanar image guidance for insertion of sacroiliac screws. Clin Orthop Relat Res 395:135–144. 10.1097/00003086-200202000-00014 11937873

[27] Smith HE, Yuan PS, Sasso R, Papadopolous S, Vaccaro AR (2006) An evaluation of image-guided technologies in the placement of percutaneous iliosacral screws. Spine 31:234–238. 10.1097/01.brs.0000194788.45002.1b 16418646

[28] Wang H, Wang F, Leong APY, Xu L, Chen X, Wang Q (2016) Precision insertion of percutaneous sacroiliac screws using a novel augmented reality-based navigation system: a pilot study. Int Orthop 40:1941–1947. 10.1007/s00264-015-3028-8 26572882

[29] Kraus MD, Krischak G, Keppler P, Gebhard FT, Schuetz UHW (2010) Can computer-assisted surgery reduce the effective dose for spinal fusion and sacroiliac screw insertion? Clin Orthop Relat Res 468:2419–2429. 10.1007/s11999-010-1393-6 PMC2919865 20521129

[30] Wähnert D, Raschke MJ, Fuchs T (2013) Cement augmentation of the navigated iliosacral screw in the treatment of insufficiency fractures of the sacrum: a new method using modified implants. Int Orthop 37:1147–1150. 10.1007/s00264-013-1875-8 PMC3664161 23553117

[31] Matityahu A, Kahler D, Krettek C, Stöckle U, Grutzner PA, Messmer P et al (2014) Three-dimensional navigation is more accurate than two-dimensional navigation or conventional fluoroscopy for percutaneous sacroiliac screw fixation in the dysmorphic sacrum. J Orthop Trauma 28:707–710. 10.1097/bot.0000000000000092 24694553

[32] Ochs BG, Gonser C, Shiozawa T, Badke A, Weise K, Rolauffs B et al (2010) Computer-assisted periacetabular screw placement: comparison of different fluoroscopy-based navigation procedures with conventional technique. Injury 41:1297–1305. 10.1016/j.injury.2010.07.502 20728881

[33] Matta JM, Saucedo T (1989) Internal fixation of pelvic ring fractures. Clin Orthop Relat Res 242:83–97. 10.1097/00003086-198905000-00009 2706863

[34] Templeman D, Schmidt A, Freese J, Weisman I (1996) Proximity of iliosacral screws to neurovascular structures after internal fixation. Clin Orthop Relat Res 329:194–198. 10.1097/00003086-199608000-00023 8769451

[35] van den Bosch EW, van Zwienen CMA, van Vugt AB (2002) Fluoroscopic positioning of sacroiliac screws in 88 patients. J Trauma 53:44–48. 10.1097/00005373-200207000-00009 12131388

[36] Bellabarba C, Schildhauer TA, Vaccaro AR, Chapman JR (2006) Complications associated with surgical stabilization of high-grade sacral fracture dislocations with spino-pelvic instability. Spine 31:S80–S88. 10.1097/01.brs.0000217949.31762.be 16685241

[37] Sagi HC, Coniglione FM, Stanford JH (2011) Examination under anesthetic for occult pelvic ring instability. J Orthop Trauma 25:529–536. 10.1097/bot.0b013e31822b02ae 21857421

[38] Gardner MJ, Routt MLC (2011) Transiliac-transsacral screws for posterior pelvic stabilization. J Orthop Trauma 25:378–384. 10.1097/bot.0b013e3181e47fad 21577075

[39] Gardner MJ, Morshed S, Nork SE, Ricci WM, Routt MLC (2010) Quantification of the upper and second sacral segment safe zones in normal and dysmorphic sacra. J Orthop Trauma 24:622–629. 10.1097/bot.0b013e3181cf0404 20871250

[40] Tile M, Pennal GF (1980) Pelvic disruption: principles of management. Clin Orthop Relat Res 151:56–64 7418324

[41] Pennal GF, Tile M, Waddell JP, Garside H (1980) Pelvic disruption: assessment and classification. Clin Orthop Relat Res 151:12–21 7418295

[42] Letournel E (1980) Acetabulum fractures: classification and management. Clin Orthop Relat Res 151:81–106 7418327

[43] Majeed S (1989) Grading the outcome of pelvic fractures. J Bone Joint Surg Br 71-B:304–306. 10.1302/0301-620x.71b2.2925751 2925751

[44] Carlson DA, Scheid DK, Maar DC, Baele JR, Kaehr DM (2000) Safe placement of S1 and S2 iliosacral screws: the vestibule concept. J Orthop Trauma 14:264–269. 10.1097/00005131-200005000-00007 10898199

[45] Stöckle U, Schaser K, König B (2007) Image guidance in pelvic and acetabular surgery: expectations, success and limitations. Injury 38:450–462. 10.1016/j.injury.2007.01.024 17403522

[46] Konrad G, Zwingmann J, Kotter E, Südkamp N, Oberst M (2010) Variabilität der Schraubenlage bei 3D-navigierter Sakrumverschraubung. Unfallchirurg 113:29–35. 10.1007/s00113-008-1546-1 19862496

[47] König B, Erdmenger U, Schröder RJ, Wienas G, Schaefer J, Pech M et al (2005) Evaluation der Bildqualität des Iso-C3D-Bildwandlers im Vergleich mit dem CT. Unfallchirurg 108:378–386. 10.1007/s00113-004-0900-1 15856147

[48] Schwarzenbach O, Berlemann U, Jost B, Visarius H, Arm E, Langlotz F et al (1997) Accuracy of computer-assisted pedicle screw placement: an in vivo computed tomography analysis. Spine 22:452–458. 10.1097/00007632-199702150-00020 9055375

[49] Wong JML, Bewsher S, Yew J, Bucknill A, de Steiger R (2015) Fluoroscopically assisted computer navigation enables accurate percutaneous screw placement for pelvic and acetabular fracture fixation. Injury 46:1064–1068. 10.1016/j.injury.2015.01.038 25683211

[50] Mendel T, Noser H, Kuervers J, Goehre F, Hofmann GO, Radetzki F (2013) The influence of sacral morphology on the existence of secure S1 and S2 transverse bone corridors for iliosacroiliac screw fixation. Injury 44:1773–1779. 10.1016/j.injury.2013.08.006 24004615

[51] Tile M (1988) Pelvic ring fractures: should they be fixed? J Bone Joint Surg Br 70-B:1–12. 10.1302/0301-620x.70b1.3276697 3276697

