# Supplementary material for: From fluoroscopy to artificial intelligence: three decades of percutaneous pelvic ring fixation—a bibliometric and science-mapping analysis (1996–2026)
Source: Arch Orthop Trauma Surg. 2026 Nov 4;146(1):396. doi: 10.1007/s00402-026-06562-3 (PMC13736322; doi:10.1007/s00402-026-06562-3)
Supplement: Supplementary file 1 [file 402_2026_6562_MOESM1_ESM.pdf]

Online Resource 1

Search strategy, verbatim search strings and stop-term list

Companion to the manuscript "From Fluoroscopy to Artificial Intelligence: Three Decades of Percutaneous Pelvic Ring Fixation. A Bibliometric and Science-Mapping Analysis (1996-2026)", submitted to Archives of Orthopaedic and Trauma Surgery. This file is cited in the Materials and methods section (Databases and search strategy; Document selection and corpus definition; Term harmonisation and stop-terms) of the main manuscript.

S1. Search strategy and verbatim search strings

Both databases were interrogated on the same date, 8 June 2026, with identical conceptual logic and database-adapted syntax. The strategy combined a pelvic-fixation-technique block (A) with a technology block (B) and a noise-exclusion block (C), and was restricted to articles, reviews and conference or proceedings papers.

S1.1 Scopus (Advanced search)

```
TITLE-ABS-KEY (
  (
    "sacroiliac screw*" OR "iliosacral screw*" OR "transsacral screw*" OR
    "transiliac screw*" OR "percutaneous pelvic" OR "pelvic ring fixation" OR
    "pelvic ring injur*" OR "posterior pelvic ring" OR "LC2 screw*" OR
    "LC-2 screw*" OR "pubic rami screw*" OR "pubic ramus screw*" OR
    "anterior column screw*" OR "sacropelvic fixation"
  )
  AND
  (
    "artificial intelligence" OR "machine learning" OR "deep learning" OR
    "neural network*" OR "navigation" OR "navigated" OR "computer-assisted" OR
    "computer assisted" OR "computer-navigated" OR "robot*" OR
    "augmented reality" OR "3D fluoroscopy" OR "intraoperative imaging"
  )
)
AND NOT TITLE-ABS-KEY (
  "degenerative" OR "scoliosis" OR "kyphosis" OR "spinal deformity" OR
  "lumbar fusion" OR "disc herniation" OR "spondylolisthesis" OR
  "acetabular fracture" OR "arthroplasty" OR "hip revision"
)
AND ( LIMIT-TO ( DOCTYPE , "ar" ) OR LIMIT-TO ( DOCTYPE , "re" )
      OR LIMIT-TO ( DOCTYPE , "cp" ) )
```

S1.2 Web of Science Core Collection (Advanced search)

```
TS=(
  (
    "sacroiliac screw*" OR "iliosacral screw*" OR "transsacral screw*" OR
    "transiliac screw*" OR "percutaneous pelvic" OR "pelvic ring fixation" OR
    "pelvic ring injur*" OR "posterior pelvic ring" OR "LC2 screw*" OR
    "LC-2 screw*" OR "pubic rami screw*" OR "pubic ramus screw*" OR
    "anterior column screw*" OR "sacropelvic fixation"
  )
  AND
  (
    "artificial intelligence" OR "machine learning" OR "deep learning" OR
    "neural network*" OR "navigation" OR "navigated" OR "computer-assisted" OR
    "computer assisted" OR "computer-navigated" OR "robot*" OR
    "augmented reality" OR "3D fluoroscopy" OR "intraoperative imaging"
  )
```

```
)
NOT TS=(
  "degenerative" OR "scoliosis" OR "kyphosis" OR "spinal deformity" OR
  "lumbar fusion" OR "disc herniation" OR "spondylolisthesis" OR
  "acetabular fracture" OR "arthroplasty" OR "hip revision"
)
```

Document types were restricted in the Web of Science Refine panel to Article, Review and Proceedings Paper. Records were exported on the search date. For the coverage corpus, Web of Science and Scopus records were merged and deduplicated in bibliometrix, by DOI and then by title and year.

Note. The exclusion phrase "acetabular fracture" was specified in the singular and, under Web of Science exact-phrase matching, did not capture records indexed under the plural "acetabular fractures". The exclusion was therefore effective against arthroplasty and isolated acetabular reconstruction, while the few retained records carrying "acetabular fractures" are mixed pelvic-ring or corridor-screw studies that matched the inclusion block.

S1.3 Records retrieved and corpora analysed

The Web of Science corpus is the primary dataset; the Scopus and merged corpora were used for cross-database robustness. The selection and deduplication sequence is shown as a flow diagram in Fig. 1 of the main manuscript.

**Corpus**	**Documents**	**Sources**
Web of Science, records exported	271	97
Web of Science (primary corpus analysed)	271	97
Scopus, records exported	765	-
Scopus, duplicate records removed on import	3	-
Scopus (robustness corpus analysed)	762	206
Web of Science and Scopus combined	1033	-
Duplicate records removed on deduplication	202	-
Web of Science + Scopus, deduplicated	831	238

S1.4 Document categories of the exported record sets

Categories are the verbatim index categories of the exported records. Eight Web of Science records fall under categories adjacent to the declared restriction (five meeting abstracts, one letter, one correction and one editorial material). These were retained so that the analysed corpus corresponds exactly to the exported record set; a sensitivity analysis excluding them is reported under Limitations in the main manuscript.

**Category**	**Web of Science**	**Scopus**
Article	230	698
Review	19	48
Article and Proceedings Paper	7	-
Proceedings Paper / Conference paper	5	19
Meeting Abstract	5	-
Letter	1	-

Correction	1	-
Editorial Material	1	-
Article and Book Chapter	1	-
Article and Early Access	1	-
**Total exported**	**271**	**765**

S2. Stop-terms and term harmonisation

The conceptual-structure analyses (keyword co-occurrence, thematic mapping, factorial analysis, trend topics) were built on the combined author-keyword and Keywords-Plus field, because author keywords alone were too sparse in this corpus to support stable thematic and trend mapping; reported keyword frequencies therefore include indexer-assigned Keywords-Plus terms as well as author keywords. The terms listed in Table S2 were treated as stop-terms and removed because they are database index, Emtree or MeSH descriptors, or publication-type tags, that carry no topical meaning and would otherwise dominate the most-frequent-word rankings. Substantive technique and technology terms (for example navigation, iliosacral screw, fluoroscopy, robotics) were retained as the topical content of the field and were not removed.

**Category**	**Stop-terms removed**
Study-population and demographic descriptors	human; humans; male; female; adult; aged; middle aged; very elderly; child
Publication-type and metadata tags	article; review; clinical article; conference paper; priority journal
Study-design descriptors	major clinical study; controlled study; retrospective study; retrospective studies; prospective study; case report; cohort analysis
Generic procedural and outcome descriptors	procedures; surgical procedures; operative; follow up; follow-up; treatment outcome; postoperative period
Parsing and placeholder artefacts	na; n/a; "SASCHA" (affiliation-field parser artefact, disregarded)

Table S2. Stop-terms excluded from the conceptual-structure analyses. The list was prespecified from inspection of the most-frequent-word output of the Scopus and merged exports.

S2.1 Orthographic and source-name harmonisation

- "iliosacral screw", "sacroiliac screw" and "SI screw" were unified to a single concept where they referred to the same implant.
- "computer-assisted", "computer assisted" and "computer-navigated" were treated as variants of computer-assisted surgery; "navigation" and "navigated" as a single navigation concept.
- "LC2" and "LC-2" (supra-acetabular or LC-II corridor) were unified.
- The two indexing forms of the journal Injury were combined for source counts; the affiliation-field artefact "SASCHA" was disregarded.
